# Adaptive Role of Cell Death in Yeast Communities Stressed with Macrolide Antifungals

**DOI:** 10.1128/mSphere.00745-21

**Published:** 2021-11-17

**Authors:** N. A. Kireeva, S. S. Sokolov, E. A. Smirnova, K. V. Galkina, F. F. Severin, D. A. Knorre

**Affiliations:** a Faculty of Bioengineering and Bioinformatics, Lomonosov Moscow State University, Moscow, Russia; b Belozersky Institute of Physico-Chemical Biology, Lomonosov Moscow State University, Moscow, Russia; University of Georgia

**Keywords:** yeast, antifungals, bioflocculation, environmental stress, macrolides, programmed cell death, stress response

## Abstract

Microorganisms cooperate with each other to protect themselves from environmental stressors. An extreme case of such cooperation is regulated cell death for the benefit of other cells. Dying cells can provide surviving cells with nutrients or induce their stress response by transmitting an alarm signal; however, the role of dead cells in microbial communities is unclear. Here, we searched for types of stressors the protection from which can be achieved by death of a subpopulation of cells. Thus, we compared the survival of Saccharomyces cerevisiae cells upon exposure to various stressors in the presence of additionally supplemented living versus dead cells. We found that dead cells contribute to yeast community resistance against macrolide antifungals (e.g., amphotericin B [AmB] and filipin) to a greater extent than living cells. Dead yeast cells absorbed more macrolide filipin than control cells because they exposed intracellular sterol-rich membranes. We also showed that, upon the addition of lethal concentrations of AmB, supplementation with AmB-sensitive cells but not with AmB-resistant cells enabled the survival of wild-type cells. Together, our data suggest that cell-to-cell heterogeneity in sensitivity to AmB can be an adaptive mechanism helping yeast communities to resist macrolides, which are naturally occurring antifungal agents.

**IMPORTANCE** Eukaryotic microorganisms harbor elements of programmed cell death (PCD) mechanisms that are homologous to the PCD of multicellular metazoa. However, it is still debated whether microbial PCD has an adaptive role or whether the processes of cell death are an aimless operation in self-regulating molecular mechanisms. Here, we demonstrated that dying yeast cells provide an instant benefit for their community by absorbing macrolides, which are bacterium-derived antifungals. Our results illustrate the principle that the death of a microorganism can contribute to the survival of its kin and suggest that early plasma membrane permeabilization improves community-level protection. The latter makes a striking contrast to the manifestations of apoptosis in higher eukaryotes, the process by which plasma membranes maintain integrity.

## INTRODUCTION

Microorganisms compete for resources, and the success of this competition depends on their ability to resist toxic compounds produced by the contenders. The resistance can be induced by multiple mechanisms. For example, cells can prevent uptake of toxic inhibitor molecules (1), actively efflux toxic compounds with plasma membrane transporters (2), or metabolize toxic molecules (3). Meanwhile, different mechanisms can cause opposite effects on surrounding cells. On the one hand, drug efflux reduces the concentration of the xenobiotic in the cytoplasm, but this does not help neighboring cells to withstand the stress. On the other hand, if a microorganism decomposes a xenobiotic, it produces a “common good” by increasing the chances of surrounding cells to survive (see reference 4 for a review). To better exploit these cooperative mechanisms, microbial cells form multicellular aggregates or biofilms. For example, bacterial cells treated with sublethal concentrations of antimicrobial peptides induce cell aggregation (5). In yeast, flocculation increases cellular tolerance to macrolide antifungal amphotericin B (AmB) and hydrogen peroxide, despite the fact that the functional flocculin allele *FLO11* decreases individual cell fitness (6). Furthermore, some yeast strains form colonies with different cell layers, where cells in the exterior layer show increased resistance to environmental stressors (7).

An extreme level of microbial cooperation is “altruistic” death, a process by which cells die to provide their neighboring cells with nutritional and environmental conditions that support their survival (8–10). The death of some cells in microbial suspension or biofilm can provide an advantage to surviving cells in different ways. For example, it has been shown that cell death in Escherichia coli mediated by the *mazF* module of *maxEF* toxin/antitoxin system can prevent the spread of the phages across the bacterial population (11). Moreover, individual E. coli cells have different bacterial toxin production rates, so some cells produce more toxins than other cells and that these cells autolyze and release toxin molecules into the medium. This toxin inhibits other bacterial strains that lack this toxin/antitoxin system (12). This strategy is considered to be an adaptive manifestation of microbial cell lysis. Furthermore, inviable cells still increase the fitness of their kin by providing them with nutrients (13, 14) or transmitting an alarm signal that causes surviving cells to preemptively adapt to stress (15, 16). In the case of pathogens, dead cells can modulate the host immune response, thereby preparing a niche for further invasion (17). Finally, dead cells can absorb toxic compounds while allowing surviving cells to continue proliferation (18). Interestingly, it has been shown that dead algal cells absorb pollutants better than living cells (19). Together, this indicates that, although dead cells cannot propagate their genes to offspring, the biochemical processes in their remnants can have a significant effect on the survival of the surrounding cells.

Meanwhile, some environmental stressors or xenobiotics induce yeast death that can be prevented by the inhibition of regulatory cascades (20–24). For example, the deletion of metacaspase or endonuclease G genes (which are homologues of mammalian apoptosis transducers) prevents yeast death induced by oxidative stress (22, 25). However, whether this genetically regulated chain of events preceding death has any adaptive role or is just a suboptimal setting of stress-response machinery is unclear.

In this study, we proposed that by undergoing cell death, yeast cells can protect their neighboring cells against some naturally occurring xenobiotics. To test this hypothesis, we screened the effects of several xenobiotics and environmental stressors on prototrophic cells while supplementing them with viable or inviable auxotrophic yeast cells. We found that dead yeast cells inhibited the cytotoxic action of macrolide antifungal AmB. Furthermore, supplementation of yeast suspension with an AmB-sensitive strain can increase the average survival of cells in this suspension upon exposure to a high concentration of AmB. Thus, our data show that, under certain conditions, decreased xenobiotic resistance in a subpopulation of cells can be beneficial for the microbial community.

## RESULTS

**FIG 1:**
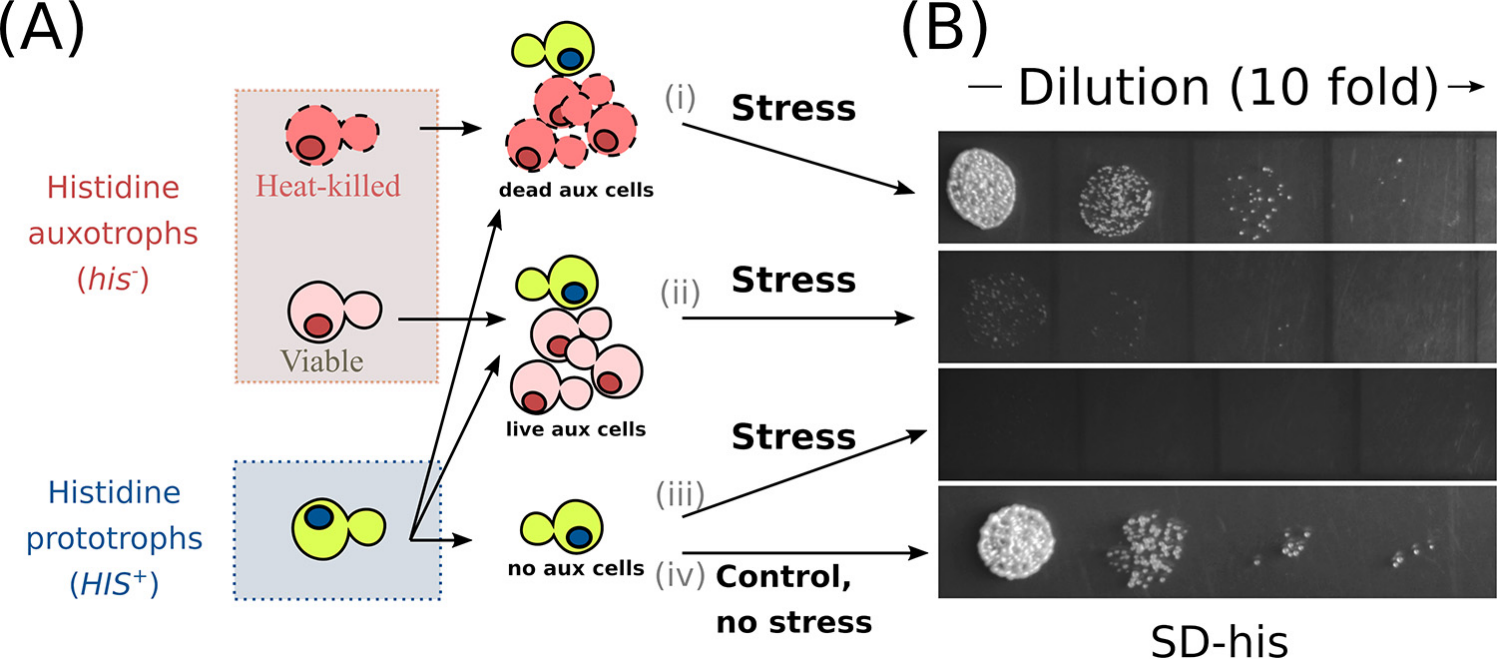
Scheme of experiment to test how an excess of viable and inviable auxotrophic cells alter the survival of prototrophic cells under various stress conditions. (A) Mixtures of histidine auxotrophic (aux) (*his*^−^) heat shock-killed cells and histidine prototrophic (*HIS*^+^) living cells (i), histidine auxotrophic (*his*^−^) control cells and histidine prototrophic (*HIS*^+^) living cells (ii), and histidine prototrophic living cells without histidine auxotrophic cells (iii) were subjected to various stressors and transferred to an SD-his agar plate. Unstressed control histidine prototrophic (*HIS*^+^) cells (iv) were plated before supplementation of stress factors. (B) Representative experiments in which AmB (7 μg/ml) was used as the stressor. In a typical experiment, we assessed 3.6 × 10^7^ cells/ml auxotrophic cells (*his*^−^) and 4 × 10^6^ cells/ml prototroph cells (*HIS*^+^).

Some stressors are better tolerated by yeast cells in dense communities than by yeast cells which are less densely dispersed, whereas other stressors kill cells irrespective of the cell suspension density. To distinguish which stressors fall into which category, we subjected prototrophic (*HIS*^+^) yeast cells (4 × 10^6^ cells/ml) to various stresses in the presence or absence of histidine auxotrophs (*his*^−^). We supplemented *HIS*^+^ cells with either living histidine auxotrophs (live aux cells in Fig. 1) or dead histidine auxotrophs (dead aux cells in Fig. 1). We performed heat shock to kill the *his*^−^ cells, because this method does not require additional procedures (e.g., centrifugation) to remove the stressor. Moreover, to assess cell suspension density effects, we tested equal and ninefold-higher cell density of histidine auxotrophs. We selected the concentration of stressors so that they kill at least 50% of *HIS*^+^ cells under the control conditions, i.e., without the addition of *his*^−^ cells. After 3 h of being under stressful conditions, cell mixtures were transferred to selective yeast synthetic dextrose agar plates without histidine (SD-his), where only *HIS*^+^ cells can grow into a colony. Finally, we calculated the number of *HIS*^+^ CFU for each tested condition and compared the effects of additionally added *his*^−^ cells.

**FIG 2:**
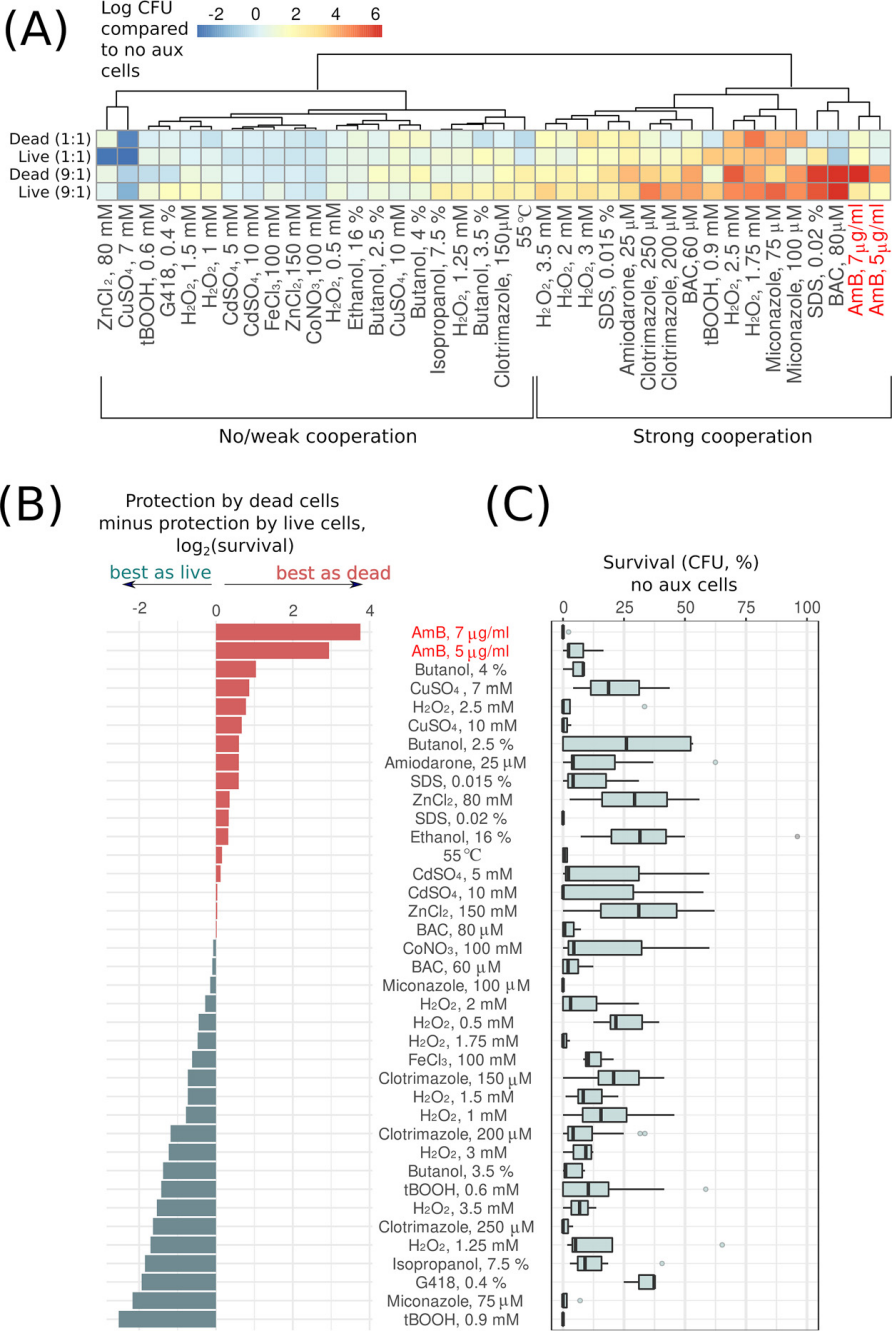
Excess of live and dead yeast cells in the suspension alleviates the lethality of some environmental stressors and antifungals. (A) Heatmap indicates relative survival of prototrophic yeast cells supplemented with different amounts of living or heat shock-killed auxotrophic cells. The values were normalized to the survival of prototrophic yeast cells treated with the same stress but not supplemented with auxotrophic (aux) cells. (B) Stressors are ranked according to whether dead or living prototroph cells contribute more or less to the survival of the auxotroph’s suspension. Zero value indicates equal contribution of dead and living cells to the community stress resistance. (C) The panel shows the survival of prototroph yeast cells under the indicated stress conditions without the addition of auxotrophic cells (no aux cells).

Supplementation of viable or dead auxotrophic cells in most cases either increased the survival of *HIS*^+^ cells or had no effect (Fig. 2A). We classified stressors depending on how strong the protective effect of dead versus living cells was by clustering the responses of four tested conditions. The analyzed types of stressors were clustered into two groups depending on whether the added auxotrophic cells helped the prototrophic cells to survive or not (Fig. 2A). Strikingly, supplementation of dead auxotrophic cells protected prototroph yeast cells against macrolide antifungal AmB much better than an equal concentration of living cells. Stressors were ordered according to the relative effectiveness of the protection achieved by dead or living cells, which revealed that dead auxotrophic cells were the most effective in protecting prototrophs from each of the tested AmB concentrations (Fig. 2B). At the same time, in our data set, we found no correlation between the relative efficacy of protection offered by dead cells and the average intensity of the stress (Fig. 2C; see also Fig. S1 in the supplemental material). Therefore, dead cells specifically increased survival in the case of AmB-induced death rather than increasing stress resistance in general.

10.1128/mSphere.00745-21.2 FIG S1 Stress intensity does not correlate with the relative effect of dead cells on the survival of the prototrophic strain. (A) Scheme of the experiment (same as in Fig. 1 and 2). The survival of prototrophic cells was calculated as the number of CFU in a suspension plated on SD-his plate after the stress divided by the number of CFU on YNB-histidine plated before the stress. (B) The relative survival of prototrophic strain supplemented with dead versus live auxotrophic cells is shown on the y axis. The survival of prototrophic cells without supplemental stress is shown on the *x* axis. The data are the same as in Fig. 2B. Each data point corresponds to the average survival of yeast cells under specific stress. Kendall’s tau = 0.1060451, z = 1.0119, *P* value = 0.3116. Download FIG S1, PDF file, 0.1 MB. Copyright © 2021 Kireeva et al. 2021 Kireeva et al. https://creativecommons.org/licenses/by/4.0/ This content is distributed under the terms of the Creative Commons Attribution 4.0 International license.

**FIG 3:**
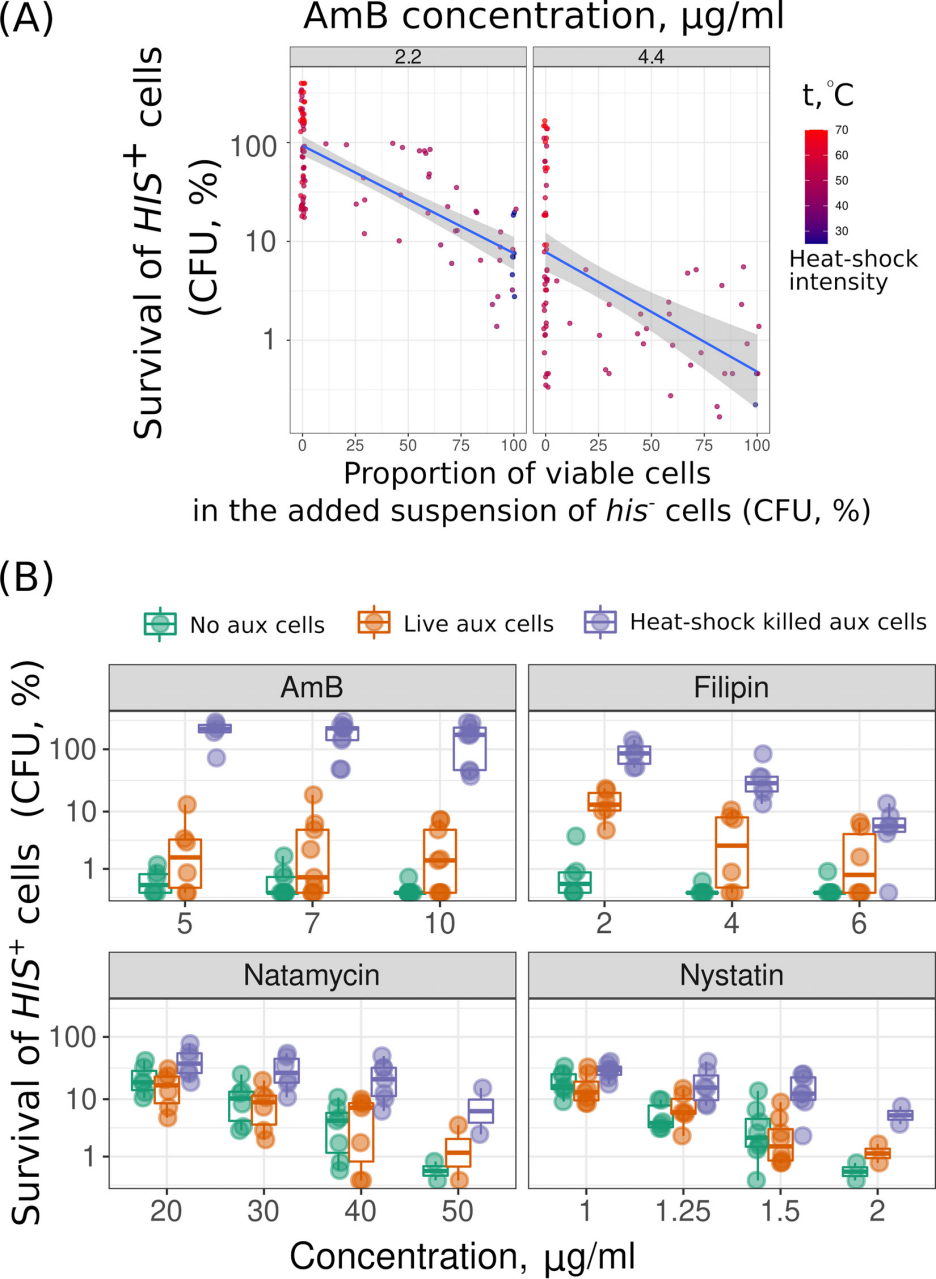
Supplementation of dead yeast cells protects yeast suspensions against macrolide antifungals better than supplementation of additional living cells. (A) Protection of prototrophic *HIS^+^* yeast cells against AmB by auxotrophic *his*^−^ cells killed by heat shock of different intensities. The protection provided by heat-shocked *his*^−^ yeast cells to prototrophic yeast cells is proportional to the percentage of inviable auxotrophic yeast cells in the suspension. With 2.2 μg/ml AmB, Kendall’s tau was 0.54 and the *P* value was 1.77 × 10^−13^; with 4.4 μg/ml AmB, tau was 0.443 and the *P* value was 1.76 × 10^−7^. To perform this experiment, we treated auxotrophic (*his*^−^ or *trp*^−^) yeast cells with different temperatures (t) (30°C to 70°C), added them to the corresponding prototrophic (*HIS*^+^ or *TRP*^+^) strain, and then subjected them to AmB for 3 h. (B) Supplementation of heat shock-killed *his*^−^ cells (heat shock-killed aux cells [violet]) but not *his*^−^ living cells (live aux cells [orange]) protected *HIS*^+^ prototrophic cells from macrolides AmB, filipin, natamycin, and nystatin.

To confirm the effect of dead cells on AmB-induced death in independent experiments, we subjected *his*^−^ yeast cells to heat shock at different intensities. As a result, we obtained yeast suspensions of *his*^−^ cells with different proportions of viable and inviable cells. We supplemented these *his*^−^ yeast suspensions to *HIS*^+^ cells and assessed the resistance of *HIS*^+^ cells to AmB. In agreement with the result of our initial survey, the proportion of *his*^−^ dead cells in the cell suspension correlated with the survival of *HIS*^+^ cells treated with AmB (Fig. 3A). Moreover, dead yeast cells protected yeast suspensions against other macrolide antifungals: filipin, natamycin, and nystatin (Fig. 3B).

**FIG 4:**
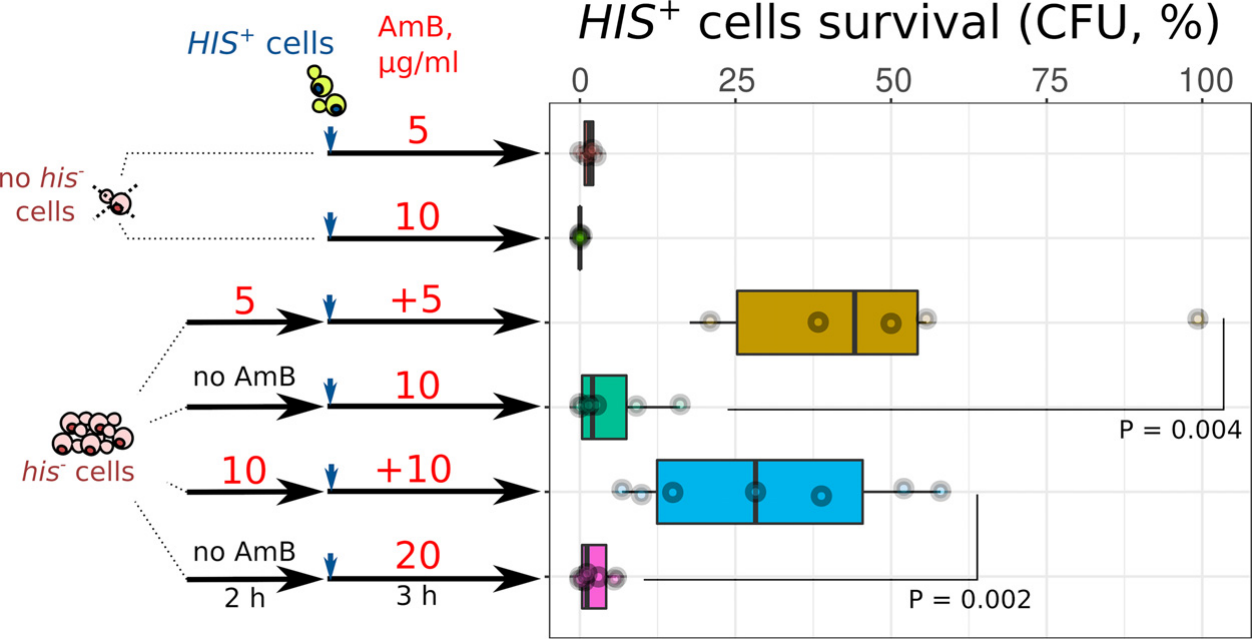
AmB-killed auxotrophic *his*^−^ yeast cells protect prototroph *HIS*^+^ yeast from AmB. Wild-type *HIS*^+^ cells (4 × 10^6^ cells/ml) were supplemented into AmB-killed *his*^−^ cells (3.6 × 10^7^ cells/ml) or untreated *his*^−^ cells (3.6 × 10^7^ cells/ml) and treated with AmB. Thereafter, we plated yeast suspensions on SD-his agar and calculated the percentage of *HIS*^+^ cells that survived AmB exposure. We calculated *P* values by the unpaired Mann-Whitney test.

To test whether the protective effect is specific to heat shock-killed cells, we performed experiments with yeast cells killed by AmB. In these experiments, we pretreated *his*^−^ auxotroph yeast cells (3.6 × 10^7^ cells/ml) with AmB for 2 h. This treatment killed roughly 65% (at 5 μg/ml AmB) and 85% (at 10 μg/ml AmB) of *his*^−^ cells. Subsequently, we supplemented either the AmB-treated suspensions or the same number of untreated *his*^−^ cells to prototrophic *HIS*^+^ yeast cells (4 × 10^6^ cells/ml) and added additional AmB (as shown in the experiment schematic in Fig. 4). After 3 h of incubation, we plated the suspension in SD-his selective medium and counted the number of CFU of *HIS*^+^ cells. Figure 4 shows that AmB-killed *his*^−^ cells protected *HIS*^+^ cells better than untreated *his*^−^ cells.

**FIG 5:**
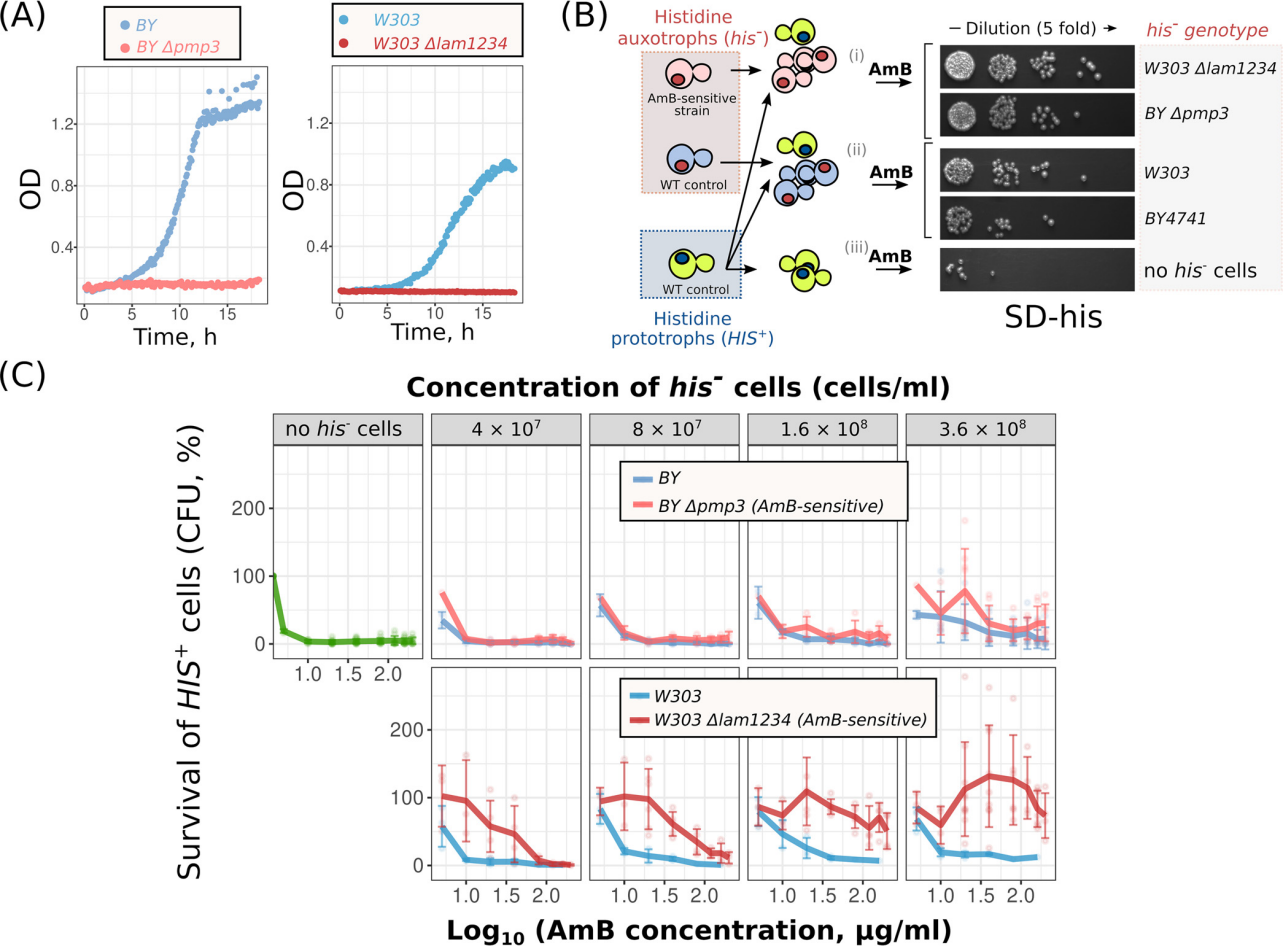
AmB-sensitive Δ*lam1* Δ*lam2* Δ*lam3* Δ*lam4* cells protect wild-type yeast cells from AmB better than the same amount of control cells. (А) Growth of Δ*pmp3*, Δ*lam1* Δ*lam2* Δ*lam3* Δ*lam4* (Δ*lam1234*), and control cells in the presence of AmB (0.8 μg/ml). (В) Scheme of the experiment. (C) Survival of wild-type *HIS*^+^ cells treated with AmB. Wild-type (*W303 or BY4741*) *HIS*^+^ cells (4 × 10^7^ cells/ml) were supplemented with an auxotrophic strain, either Δ*pmp3 his*^−^ or control *BY4741* *his*^−^ cells (top panel) or either Δ*lam1* Δ*lam2* Δ*lam3* Δ*lam4* (Δ*lam1234*) or *W303* control (bottom panel). Concentrations of auxotrophic cells are indicated in the top row of the panel.

Given that dead cells showed higher efficiency in protecting remaining surviving cells from macrolides, we reasoned that the supplementation of wild-type yeast suspensions with AmB-hypersensitive cells could increase the proportion of surviving cells. To test this hypothesis, we took Δ*pmp3* and Δ*lam1* Δ*lam2* Δ*lam3* Δ*lam4* strains that were previously demonstrated to be sensitive to AmB (26, 27). We confirmed that AmB inhibited the growth of these strains at low concentrations, which did not prevent the growth of the parental strains (Fig. 5A). Importantly, we have found that Δ*lam1* Δ*lam2* Δ*lam3* Δ*lam4* cells lose the ability to form colonies after the addition of AmB much more rapidly than cells of the parental strain (Fig. S2). Then, we exposed yeast suspensions composed of the wild-type histidine prototrophic strain (*HIS*^+^, cell density 4 × 10^7^ cells/ml) and either of these strains to different concentrations of AmB for 3 h. For a control, we supplemented the wild-type histidine prototroph with the parental auxotrophic strain (*his*^−^, see the experimental design scheme in Fig. 5B). We found that additional supplementation of the Δ*lam1* Δ*lam2* Δ*lam3* Δ*lam4 his*^−^ cells protected the wild-type yeast *HIS*^+^ strain from a high concentration of AmB (Fig. 5C) and filipin (Fig. S3). Simultaneously, the control *his*^−^ cells were inefficient in protecting *HIS*^+^ prototrophs from AmB (Fig. 5C). The protection effect increased with the increase in auxotroph cell density. In the case of Δ*lam1* Δ*lam2* Δ*lam3* Δ*lam4 his*^−^ cells, the protection of *HIS*^+^ was already pronounced at a cell density of 4 × 10^7^. Δ*pmp3 his*^−^ cells increased AmB resistance of *HIS*^+^ cells when added at a concentration nine times that of *HIS*^+^ cells (3.6 × 10^8^ cells/ml) but only marginally.

10.1128/mSphere.00745-21.3 FIG S2 The death rate dynamics of *WT* and Δ*lam1* Δ*lam2* Δ*lam3* Δ*lam4* cells treated with AmB (3 to 4.5 μg/ml; the concentration is indicated above each graph). Download FIG S2, PDF file, 0.06 MB. Copyright © 2021 Kireeva et al. 2021 Kireeva et al. https://creativecommons.org/licenses/by/4.0/ This content is distributed under the terms of the Creative Commons Attribution 4.0 International license.

10.1128/mSphere.00745-21.4 FIG S3 Survival of WT *HIS^+^* cells treated with AmB. WT *HIS^+^* cells (2 × 10^7^ cells/ml) were supplemented with *his*^−^ Δ*lam1* Δ*lam2* Δ*lam3* Δ*lam4* (*W303* Δ*lam1234*) strain or *his*^−^ *W303* control. The concentrations of auxotrophic cells are indicated above the graphs. Download FIG S3, PDF file, 0.1 MB. Copyright © 2021 Kireeva et al. 2021 Kireeva et al. https://creativecommons.org/licenses/by/4.0/ This content is distributed under the terms of the Creative Commons Attribution 4.0 International license.

**FIG 6:**
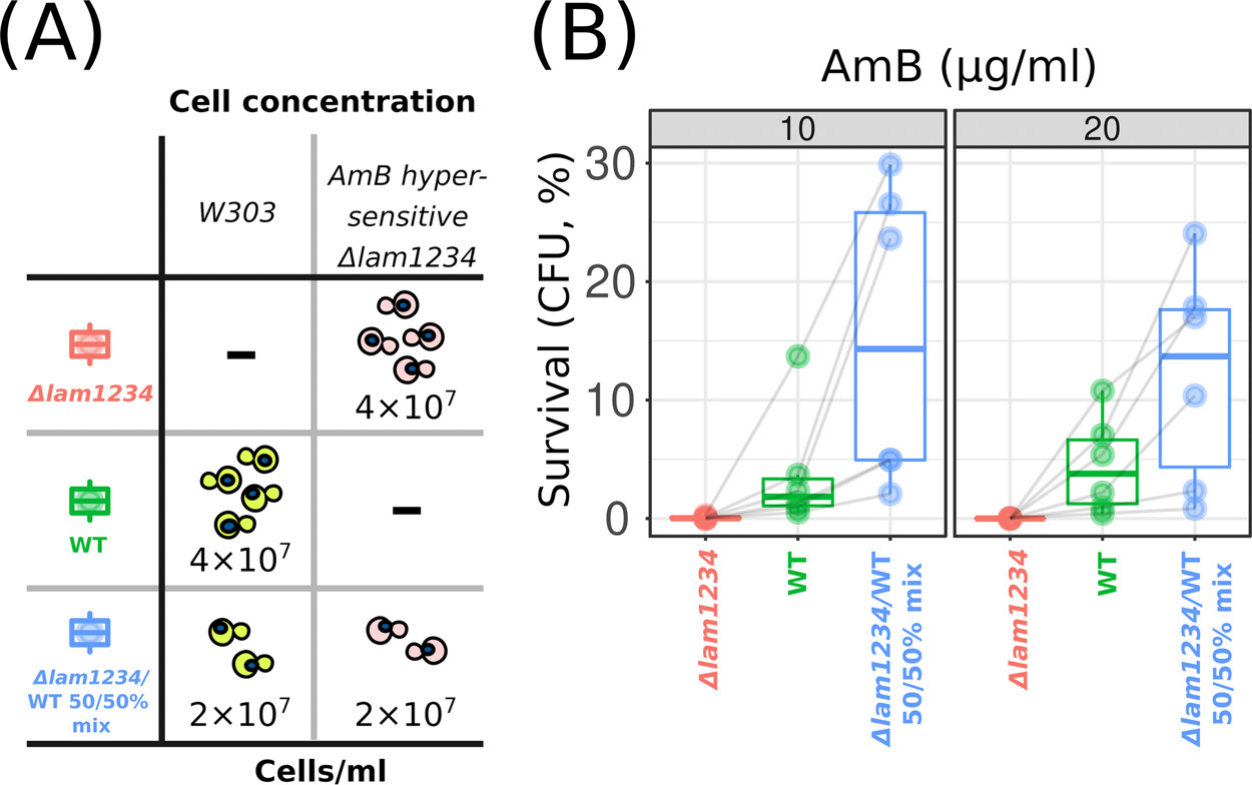
Cell mixture of WT and AmB-hypersensitive cells survive AmB better than homogenic WT cells. (A) Scheme of the experiment and figure legend. In all cases, we equalized the final concentration of cells in the testing tubes. Numbers designate the final concentration of cells per milliliter. (B) Average yeast cell survival in the wild-type (*W303*), Δ*lam1* Δ*lam2* Δ*lam3* Δ*lam4* (Δ*lam1234*), and the wild-type/Δ*lam1234* mixed suspensions treated with 10 or 20 μg/ml of AmB. In these experiments, we assessed cell survival by calculating the number of CFU in YPD plates after 3 h of AmB treatment (e.g., we did not distinguish the strain of surviving cells from the mixed suspensions). Shaded gray lines connect data points from separate day experiments. *P* = 0.027 according to paired Wilcoxon signed rank test for a comparison of the wild-type/Δ*lam1234* mixed suspension with the wild-type suspension.

Strikingly, we found that the suspension when consisting of equal proportions of Δ*lam1* Δ*lam2* Δ*lam3* Δ*lam4* cells (2 × 10^7^ cells/ml) and control cells (2 × 10^7^ cells/ml) produced more CFU on rich medium than the control cell suspension (4 × 10^7^ cells/ml) if treated with the same concentration of AmB (Fig. 6). This result means that each AmB-sensitive cell that was killed by AmB saved more than one control cell with the wild-type AmB resistance.

**FIG 7:**
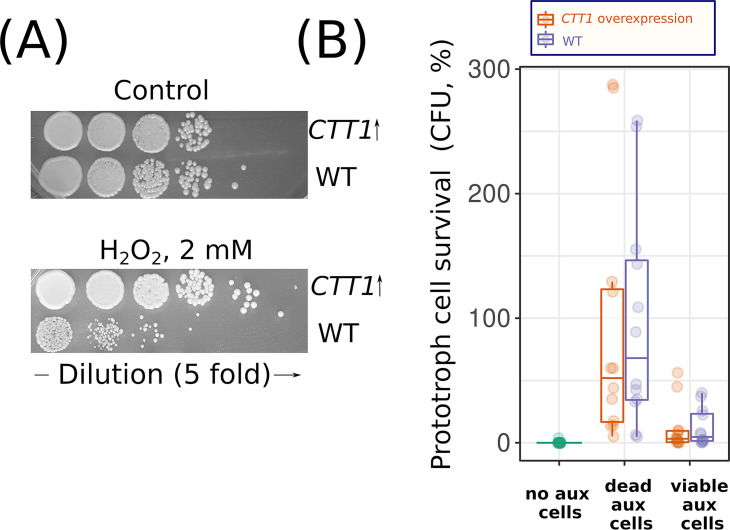
Overexpression of *CTT1* in *trp*^−^ cells provides no increase in AmB resistance for *TRP*^+^ cells in the same suspension. (A) Overexpression of *CTT1* provides resistance to hydrogen peroxide (3 h of incubation times). To increase *CTT1* expression, we incubated the P_GAL_-*CTT1* strain in galactose-containing rich medium (YPGal) overnight. (B) Control *trp*^−^ or P_GAL_-*CTT1 trp*^−^ cells were supplemented to *TRP*^+^ cells. The *trp*^−^ cells were killed in advance with heat shock (dead aux cells) or remained untreated (viable aux cells). Cell mixtures were treated with 7 μg/ml AmB; the incubation time with AmB was 3 h. Otherwise, the experimental design was as described in the legend to Fig. 1 and in Materials and Methods.

We tested two possibilities to obtain insight into the mechanisms by which dead cells can protect living cells. First, it was shown previously that AmB toxicity is mediated by secondary oxidative stress (28) and can be alleviated by the supplementation of antioxidants (29). We suggested that dead cells can release catalase from their cytoplasm into the incubation media and in this way protect living cells from AmB. To test this possibility, we put a genomic copy of cytoplasmic catalase gene *CTT1* under the regulation of a P_GAL_ promoter (see Materials and Methods) and overexpressed it by growing yeast in a galactose-containing medium. P_GAL_-*CTT1 trp*^−^ catalase overexpression cells showed increased resistance to hydrogen peroxide (Fig. 7A) but provided no increase in survival to corresponding prototrophic *TRP*^+^ cells subjected to amphotericin (Fig. 7B).

**FIG 8:**
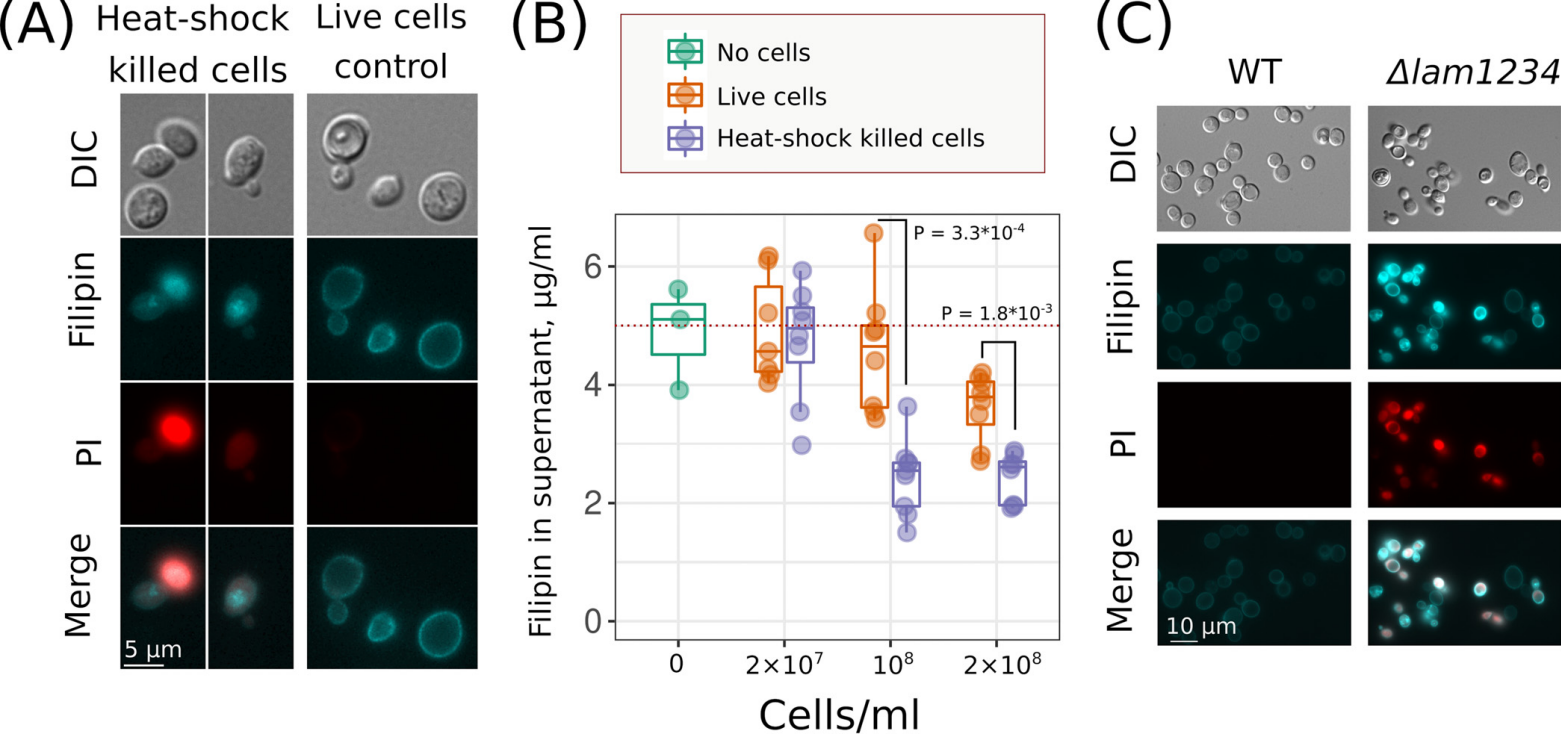
Dead yeast cells absorb macrolide filipin with intracellular compartments. (A) Different localization of the filipin signal in heat shock-killed and live control cells. DIC, differential interference contrast; PI, propidium iodide. (B) Heat shock-killed cells absorb more filipin compared to viable control cells. Suspension of yeast cells was supplemented with filipin (5 μg/ml) and then centrifuged. Integral fluorescence spectra in the supernatant were measured. *P* values were calculated according to the unpaired Mann-Whitney test. (C) Filipin staining induced permeabilization of Δ*lam1* Δ*lam2* Δ*lam3* Δ*lam4* (*lam1234*) strain but not the wild-type strain. Yeast cells were treated with filipin (5 μg/ml; incubation time, 5 min).

The second possibility we considered was that dead yeast cells absorb macrolide from the medium and therefore decrease the amount of antifungals bound to the membranes of living cells. To test this, we stained the control and heat shock-killed cells with filipin, which has a high fluorescent yield and is often used for visualizing sterol-rich membranes in yeast cells (27, 30). Figure 8A shows that while control cells were stained only at the periphery, the heat shock-killed cells exhibited intracellular compartment staining. To quantify the absorption of filipin by dead and living cells, we incubated the yeast suspensions with filipin (5 μg/ml), centrifuged the suspension, and measured the residual fluorescence in the supernatant (see Materials and Methods for details). The addition of dead cells to a filipin-containing incubation medium decreased filipin fluorescence in the supernatant (Fig. 8B). A suspension of dead yeast cells (10^8^ cells/ml) absorbed an average filipin concentration of 2.55 μg/ml. At the same time, the same concentration of living control cells decreased the concentration of filipin in the supernatant by only 0.42 μg/ml; therefore, a dead cell killed by heat shock absorbs approximately six times more macrolide filipin than a living cell.

Next, we treated the wild-type cells and Δ*lam1* Δ*lam2* Δ*lam3* Δ*lam4* mutant cells with filipin. In these cells, we analyzed filipin intracellular localization. To test the integrity of the plasma membrane, we used propidium iodide, which is commonly used to detect dead cells. Figure 8C shows that 5 min of treatment of yeasts with filipin induced propidium iodide staining and intracellular filipin localization in Δ*lam1* Δ*lam2* Δ*lam3* Δ*lam4* cells but not in the control *W303* cells.

## DISCUSSION

Cooperation among neighboring cells can increase their resistance against some stressors but can be futile against the others. In the case of xenobiotics, hydrophobicity of the molecule is one of the basic factors determining the efficiency of cellular cooperation against it. Indeed, xenobiotics accumulating in cell membranes and lipid droplets can be depleted from media if there are excess cells and limited sources of xenobiotics. Accordingly, our survey of stressors (Fig. 2A) showed that supplementation of additional auxotroph cells increased the survival of prototroph cells toward hydrophobic azole antifungals (e.g., miconazole) and surfactants (e.g., benzalkonium chloride [BAC]) but did not alter their survival in the presence of heavy metals (e.g., CdSO_4_) or fusel alcohols (e.g., butanol). Additional yeast cells in suspensions also increased resistance to high concentrations of hydrogen peroxide (Fig. 2A), which was probably due to the contribution of cellular antioxidant systems to the decomposition of hydrogen peroxide.

Meanwhile, we found that only nonviable cells provide significant protection against macrolide antifungals AmB, filipin, and, to a lesser extent, natamycin and nystatin (Fig. 3B). All of these compounds are produced by different species of *Streptomyces*—a widespread Gram-positive soil filamentous bacteria (31). The biosynthesis of some of these compounds is species specific: nystatin (Streptomyces noursei), AmB (Streptomyces nodosus), and others that are less specific, such as natamycin (32). Macrolide antifungals bind sterol-rich membranes, induce their conductance, and/or deplete membrane sterol from the membrane while disturbing its vital properties (33, 34). At the same time, the interaction of macrolide with the membranes determines how efficiently it can be absorbed by dead cells. For example, filipin shows lower specificity toward ergosterol-rich membranes than natamycin and nystatin (35). Therefore, filipin should be more effectively absorbed by the organellar membranes than natamycin and nystatin. Meanwhile, pretreatment of AmB with free ergosterol decreases its antifungal activity (increases MIC) to a greater extent than it decreases the activity of nystatin and natamycin (36). We suggest that the combination of these factors explains the fact that dead cells protect living cells from AmB and filipin better than from other tested macrolides.

The amphiphilic nature of the macrolides suggests that they cannot passively diffuse across the membrane; moreover, cell walls additionally restrain macrolide AmB absorption by yeast cells (37). Therefore, in the suspension of live cells, macrolides interact primarily with the outer leaflet of plasma membranes. Although the plasma membrane contains more sterol than the membranes of other organelles (38), its surface area is much smaller than the integral surface of the cell membranes of permeabilized cells. Moreover, some studies suggest that sterols are unevenly distributed within plasma membranes, with a major amount of sterol being available only from the inner (cytosol) leaflet of the lipid bilayer (39). For example, in yeast, only 20% of fluorescent dehydroergosterol can be quenched by impermeable fluorescent quenchers, as efficient quenching requires the disruption of the plasma membrane integrity (40). Intriguingly, in our recent study, we found that the deletion of sterol-transporting LAM genes increase filipin staining of yeast cells in both the plasma membrane and intracellular compartments (27). The high sensitivity of the LAM-deficient strain to filipin (Fig. 8C) suggests that intense plasma membrane staining in these experiments can be explained by filipin binding to the inner leaflet of dead yeast plasma membrane rather than an increase in the sterol concentration. Therefore, permeabilization of yeast cells can expose additional macrolide-binding sites.

Given that permeabilized yeast cells absorb more macrolide antifungals than living yeast cells, a yeast community (e.g., dense suspension or colony) can benefit from early permeabilization of plasma membranes in a subpopulation of cells. This occurs in striking contrast to the apoptosis of mammalian cells, which maintains plasma membrane integrity to prevent the release of proinflammatory factors (41). Meanwhile, the metazoa in some cases rely on inflammation upregulation. Accordingly, during pyroptotic cell death of mammalian cells, plasma membrane rupture is facilitated by small plasma membrane proteins gasdermin D (42) and NINJ1 (43). Therefore, we speculate that the physiological scenarios of programmed cell death in yeast should be either homologous or analogous to metazoa programmed cell death mechanisms during early plasma membrane rupture.

Whether clonal microbial populations are heterogeneous is determined by the individual cells’ stress resistance phenotype (44, 45). This cell-to-cell heterogeneity arises from transcriptional noise, cell cycle-mediated differences, and in the case of budding yeasts, division asymmetry (46, 47). An increase in the variance of stress resistance phenotypes among individual cells in the population can improve the survival of clonal lineages through repetitive severe stresses (48). Our data extend these observations by exemplifying that improved survival in a suspension can be achieved by an increase in the variance of macrolide tolerance, even if this increase is associated with a decrease in the average tolerance. Indeed, Fig. 5 and 6 show that the substitution of the control cells in a suspension with AmB-sensitive cells increases overall survival. We suggest that macrolide resistance heterogeneity can be an adaptive trait that evolves to help cellular clonal communities withstand a high concentration of macrolides.

## MATERIALS AND METHODS

### Yeast strains, growth medium, and reagents.

We used standard yeast rich and synthetic mediums described by Sherman (49). Yeast strains used in the study are listed in Table S1 in the supplemental material. To generate a strain with *CTT1* overexpression, we substituted the native *CTT1* promoter with a gene cassette containing the P_GAL_ promoter and a marker gene. To produce the cassette, we used the PCR-based approach described by Longtine et al. (50) using pFA6aHIS3MX6-PGAL1 plasmid as matrix DNA and the following gene-specific primers: CTT1-F (5′-CTCAATCTTGTCGTTACTTGCCCTTATTAAAAAAATCCTTCTCTTGTCTCGAATTCGAGCTCGTTTAAAC-3′) and CTT1-R (5′-TTTTTACCGAACACGTTCATTTGTGAAGCTGAGCTGATTGATCTTATTGGCATTTTGAGATCCGGGTTTT-3′). Primers CTT1-test-F (5′-AATGATGAGTACGTGCCCGAT-3′) and CTT1-test-R (5′-CACCTTCAAGAGGTTTAGGAA-3′) were used to validate the correct clones due to the size of the PCR product.

10.1128/mSphere.00745-21.1 TABLE S1 Strains used in the study. Download Table S1, DOCX file, 0.01 MB. Copyright © 2021 Kireeva et al. 2021 Kireeva et al. https://creativecommons.org/licenses/by/4.0/ This content is distributed under the terms of the Creative Commons Attribution 4.0 International license.

### Testing how an excess of live and dead auxotrophic cells alters the survival of prototrophic cells under various stress conditions.

The cells were incubated overnight in 50-ml tubes with 5 ml liquid synthetic dextrose medium with all amino acids (SD) at a cell density of 4 × 10^6^ to 8 × 10^6^ cells/ml (logarithmic growth stage). The cells were collected by centrifugation (700 × *g*, 5 min), and the medium was removed. The cells of histidine auxotrophic (*his*^−^) and histidine prototrophic (*HIS*^+^) strains were resuspended in a synthetic dextrose medium without histidine (SD-his) at a cell density of 4 × 10^7^ cells/ml. The histidine auxotrophic (*his*^−^) cells were divided equally into two tubes, and one half was then killed by heating at 55°C for 30 min. We plated the *his*^−^ suspension on yeast extract-peptone-dextrose (YPD) plates before and after heat shock treatment to ensure that less than 5% of cells survived heat shock. Then, *HIS*^+^ cells and *his*^−^ cells (live or dead) were mixed at a density of 4 × 10^6^ cells/ml at a ratio of 1:0, 1:1, or 1:9. The experiment was performed in a 96-well plate (Eppendorf; catalog no. 0030730011) with a final cell mixture volume of 200 μl per well.

Then, xenobiotics or other stress factors were added, and the plate was incubated at 30°C and 500 rpm for 3 h. In experiments with metal salts, we increased the incubation time to 20 h to ensure that more than 50% of *HIS*^+^ cells were killed under the control conditions. Each sample was diluted 75 times and suspended; then, 5 μl of the diluted suspension was transferred onto SD-his agar plates. CFU were counted in 24 to 48 h (see the scheme of the experiment in Fig. 1A and experimental results in Fig. 2A, Fig. 3B, and Fig. 7). Figure 2 includes some experiments performed with fourfold-lower cell density. Moreover, in some of the experiments shown in Fig. 2, we used the *TRP*^+^/*trp*^−^ prototroph/auxotroph pair using a protocol that was similar to the protocol for the *HIS*^+^/*his*^−^ pair.

We did not consider the possibility of histidine or tryptophan auxotrophy reversion in our experiments, given that no single colony had formed in the SD-his and SD-trp plates when supplemented with the corresponding prototrophic strain cells. Uncropped photographs of the agar plate with spots from Fig. 1 and 5 are presented in Fig. S5 in the supplemental material.

10.1128/mSphere.00745-21.6 FIG S5 Original uncropped photographs of yeast plates from Fig. 1B (left) and Fig. 5B (right). Download FIG S5, PDF file, 1.2 MB. Copyright © 2021 Kireeva et al. 2021 Kireeva et al. https://creativecommons.org/licenses/by/4.0/ This content is distributed under the terms of the Creative Commons Attribution 4.0 International license.

To generate a set of auxotrophic cells with various proportions of dead cells, the tryptophan auxotrophic strain (*trp*^−^) *W303* was exposed to different temperatures (30°C to 70°C) for 30 min. The survival rate of auxotrophic cells was determined as the ratio of CFU after heat shock before heat shock. Prototrophic *TRP*^+^ and auxotrophic *trp*^−^ cells (containing various proportions of dead cells) were mixed at a density of 1 × 10^6^ cells/ml at a ratio of 1:9. The experiment was performed in a 96-well plate with a final cell mixture volume of 200 μl per well. AmB at a concentration of 2.2 μg/ml or 4.4 μg/ml was added to the resulting mixtures; then, the mixtures were incubated for 3 h at 30°C and 500 rpm. Each sample was diluted 75 times and suspended; then, 5 μl of the diluted suspension was transferred onto SD-trp agar plates. CFU were determined after 2 days of growth at 30°C (see the experimental results in Fig. 3A).

### Testing the ability of AmB-killed auxotrophic yeast cells to protect prototrophic *HIS*^+^ yeast from AmB.

Histidine auxotrophic (*his*^−^) cells were resuspended in liquid SD-his medium at a concentration of 4 × 10^7^ cells/ml. One part of the *his*^−^ cell suspension was pretreated with 5 μg/ml or 10 μg/ml AmB for 2 h (see schematic in Fig. 4); another part was left untreated. Then, we supplemented these suspensions with *HIS*^+^ cells. We added an additional AmB (5 μg/ml or 10 μg/ml) into samples with pretreated *his*^−^ cells. Into samples with untreated *his*^−^ cells, we added AmB to the final concentration (10 μg/ml or 20 μg/ml). For an auxotrophic cell-free control, we inoculated *HIS*^+^ cells in the same volume of SD-his and treated them with 10 μg/ml or 20 μg/ml AmB. Cell mixtures were incubated in 96-well microplates for 3 h at 30°C and shaken at 500 rpm. Then, each sample was diluted and transferred onto SD-his agar plates. CFU were counted in 24 to 48 h (see the scheme of the experiment and results in Fig. 4).

### Growth kinetics.

Exponentially growing cells were diluted to an optical density at 550 (OD_550_) of 0.2 and inoculated into a 48-well plate (Greiner). Plates were incubated at 30°C in a spectrophotometer (SpectrostarNANO) with the following settings: orbital shaking at 500 rpm for 30 s before measurements; OD_550_ measurements were performed at 5-min intervals (Fig. 5A).

### Testing the ability of AmB-hypersensitive Δ*pmp3* and Δ*lam1* Δ*lam2* Δ*lam3* Δ*lam4* strains to protect wild-type cells from AmB.

The cells were incubated overnight in 50-ml tubes with 5 ml of liquid SD to a final cell concentration of 4 × 10^6^ to 8 × 10^6^ cells/ml (logarithmic growth stage). The cells were collected via centrifugation (700 × *g*, 5 min) and resuspended in SD-his. Then, *HIS*^+^ cells (2 × 10^7^ cells/ml) were mixed with *his*^−^ AmB-hypersensitive cells: Δ*pmp3*, Δ*lam1* Δ*lam2* Δ*lam3* Δ*lam4*, or parental strain (*BY4741* or *W303*) in different proportions (see Fig. 5 legend). The experiment was performed in a 96-well plate with a final cell mixture volume of 200 μl per well. Then, different concentrations of AmB (from 5 to 120 μg/ml) were added, and the plate was incubated at 30°C and 500 rpm for 3 h. Each sample was diluted 75 times and suspended; then, 5 μl of the diluted suspension was transferred onto plates with SD-his. CFU were counted after 24 to 48 h (Fig. 5B and C).

For the experiment shown in Fig. 6, the wild-type and AmB-hypersensitive Δ*lam1* Δ*lam2* Δ*lam3* Δ*lam4* cells from the logarithmic growth stage were collected using centrifugation (700 × *g*, 5 min) and resuspended in SD medium at 4 × 10^7^ cells/ml. Then, each strain and their mixture in a 1:1 ratio was treated with 10 or 20 μg/ml of AmB and incubated at 30°C and 500 rpm for 3 h. Subsequently, the suspensions were diluted and transferred onto YPD plates, where cells grew irrespective of their prototrophic markers. CFU were counted in 24 to 48 h (Fig. 6).

### Fluorescence microscopy.

We resuspended wild-type and mutant yeast cells in 50 mM potassium phosphate buffer to a final concentration of 5 × 10^7^ cells/ml and supplemented the suspension with filipin (filipin complex from Streptomyces filipinensis; Sigma catalog no. F9765) to a final concentration of 5 μg/ml. After 5 min of incubation, filipin was removed from the medium by centrifugation, and cells were supplemented with propidium iodide (Thermo Fisher Scientific, catalog no. P3566; final concentration, 1 μg/ml). To photograph cells, we used an Olympus BX41 microscope with a U-MNU2 filter (excitation wavelength, 360 to 370 nm; beam splitter filter, 360 to 370 nm; emission, > 420 nm) for filipin and U-MNG2 filter (excitation, 530 to 550 nm; beam splitter filter, 570 nm; emission, >590 nm) for propidium iodide. Photographs were taken with a DP30BW charged-coupled-device camera (Fig. 8A and C).

### Filipin absorption experiments.

Cells were grown overnight on SD agar plates and then resuspended in 50 mM potassium phosphate buffer, pH 5.5, to a final cell concentration of 2 × 10^7^, 1 × 10^8^, or 2 × 10^8^. Filipin was added to a final concentration of 5 μg/ml. After 5-min incubation, the cells were centrifuged, and supernatant was transferred to a 96-well plate. Fluorescence of unabsorbed filipin was analyzed using Fluoroskan Ascent (excitation, 355 nm; emission, 460 nm). The filipin calibration curve, as shown in Fig. S4, revealed the linearity of the tested concentration of filipin. The results of the absorption experiment are shown in Fig. 8B.

10.1128/mSphere.00745-21.5 FIG S4 Calibration curve for filipin. Fluorescence was analyzed using Fluoroskan Ascent (excitation, 355 nm: emission, 460 nm). Download FIG S4, PDF file, 0.02 MB. Copyright © 2021 Kireeva et al. 2021 Kireeva et al. https://creativecommons.org/licenses/by/4.0/ This content is distributed under the terms of the Creative Commons Attribution 4.0 International license.

### Data analysis.

We analyzed data and generated the figures with R tidyverse libraries (51). A heatmap was generated with the pheatmap R package (version 1.0.12) with default parameters that use maximum linkage clustering. Where possible, we have shown individual data points and provided connections between data points obtained from the same experiment.
