# Supplementary figures and images for: Adaptive Role of Cell Death in Yeast Communities Stressed with Macrolide Antifungals
Source: mSphere. 2021 Nov 17;6(6):e00745-21. doi: 10.1128/mSphere.00745-21 (PMC8597739; doi:10.1128/mSphere.00745-21)

(A)

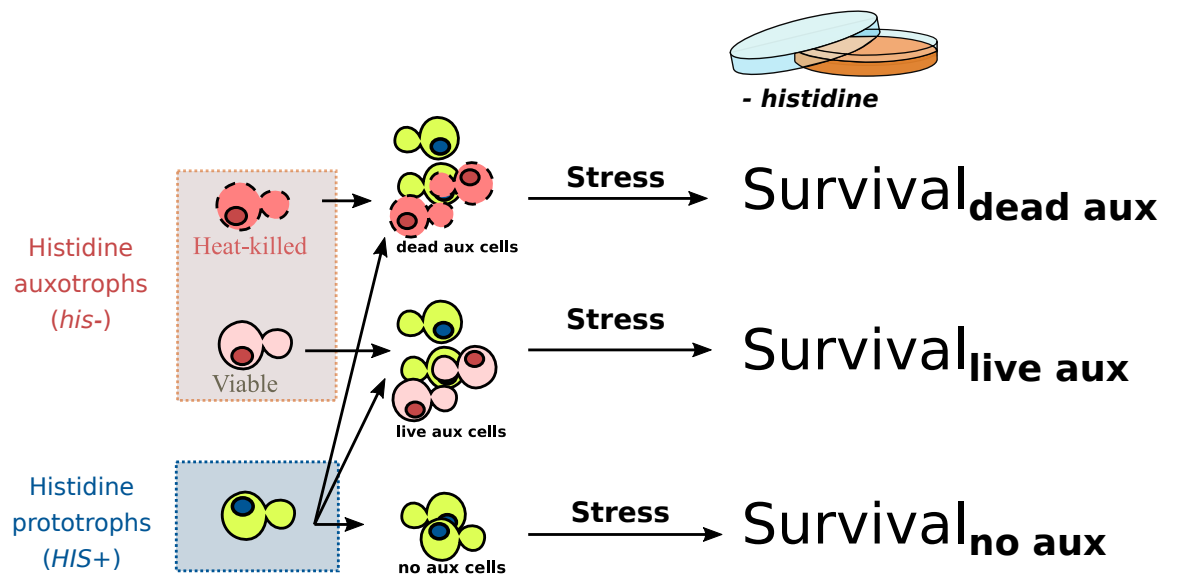

(B)

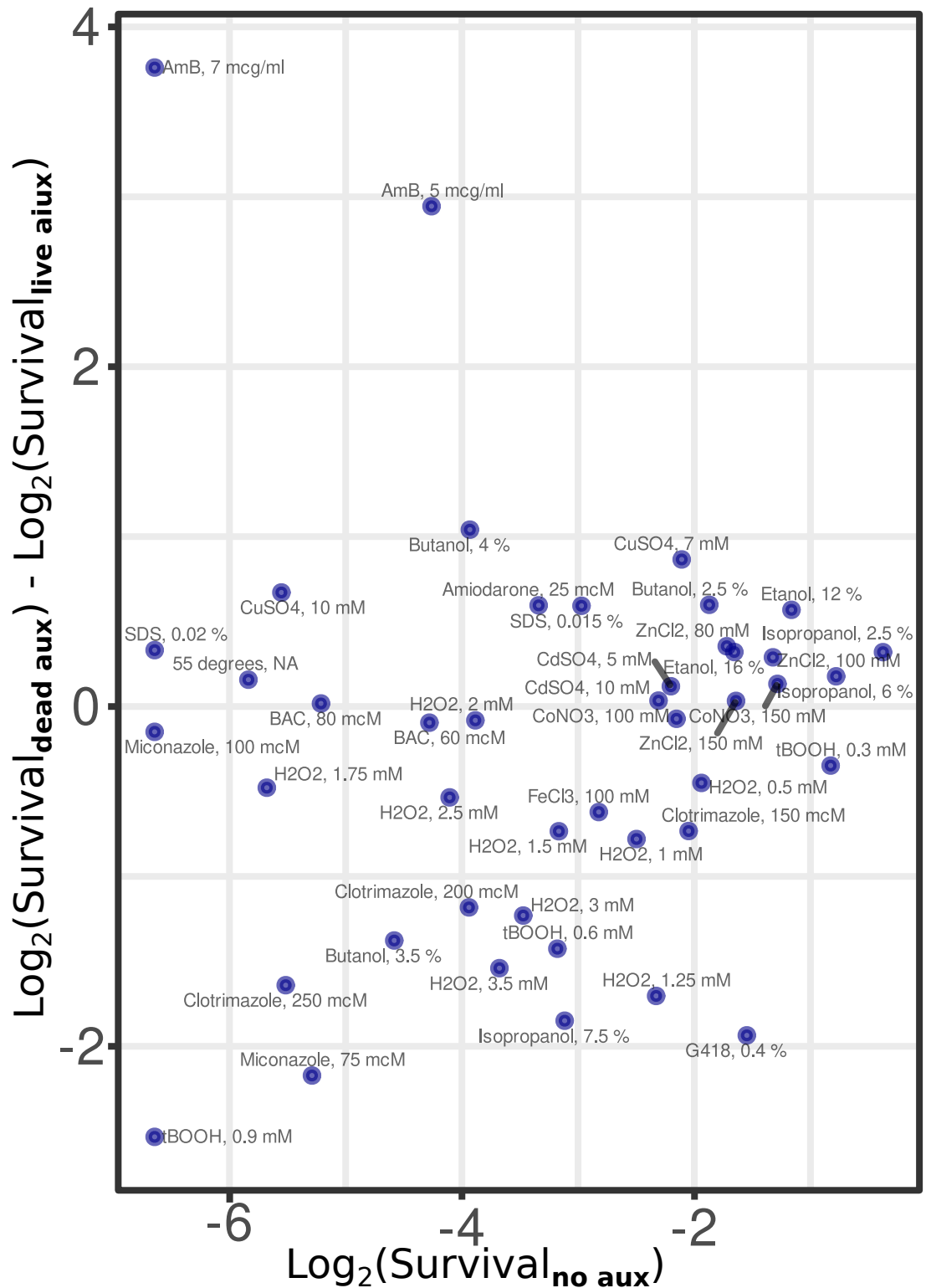

Supplement: FIG S1 [file msphere.00745-21-sf001.pdf]

# AmB ( $\mu\text{g/ml}$ )

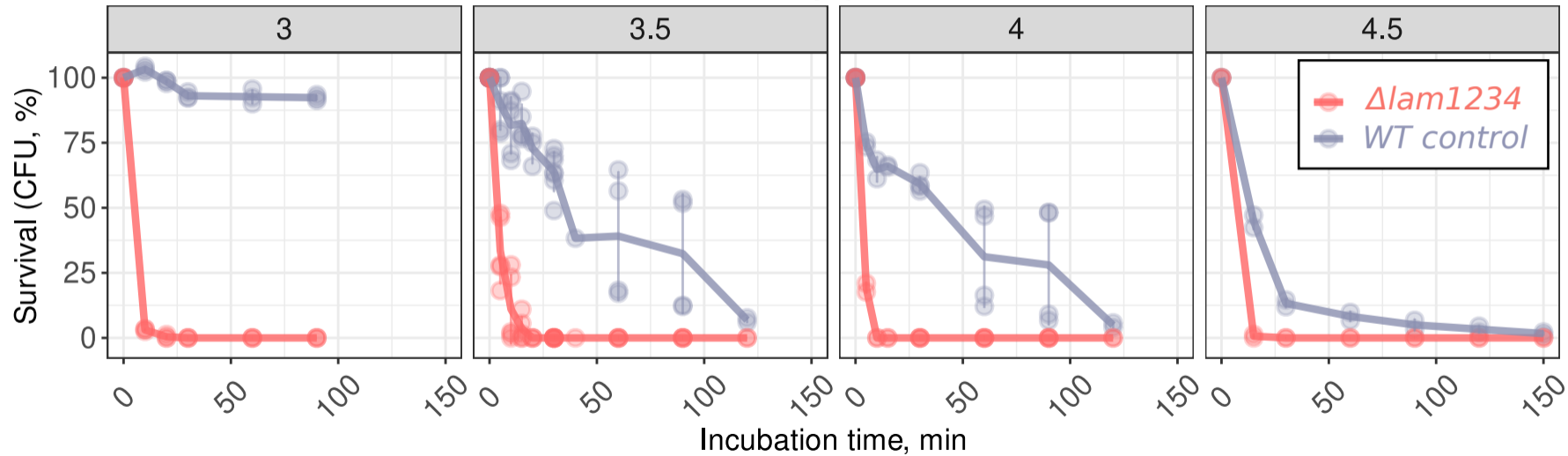

Supplement: FIG S2 [file msphere.00745-21-sf002.pdf]

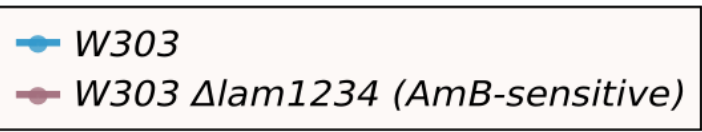

Cell density of histidine auxotrophes cells ( $10^7$  cells/ml)

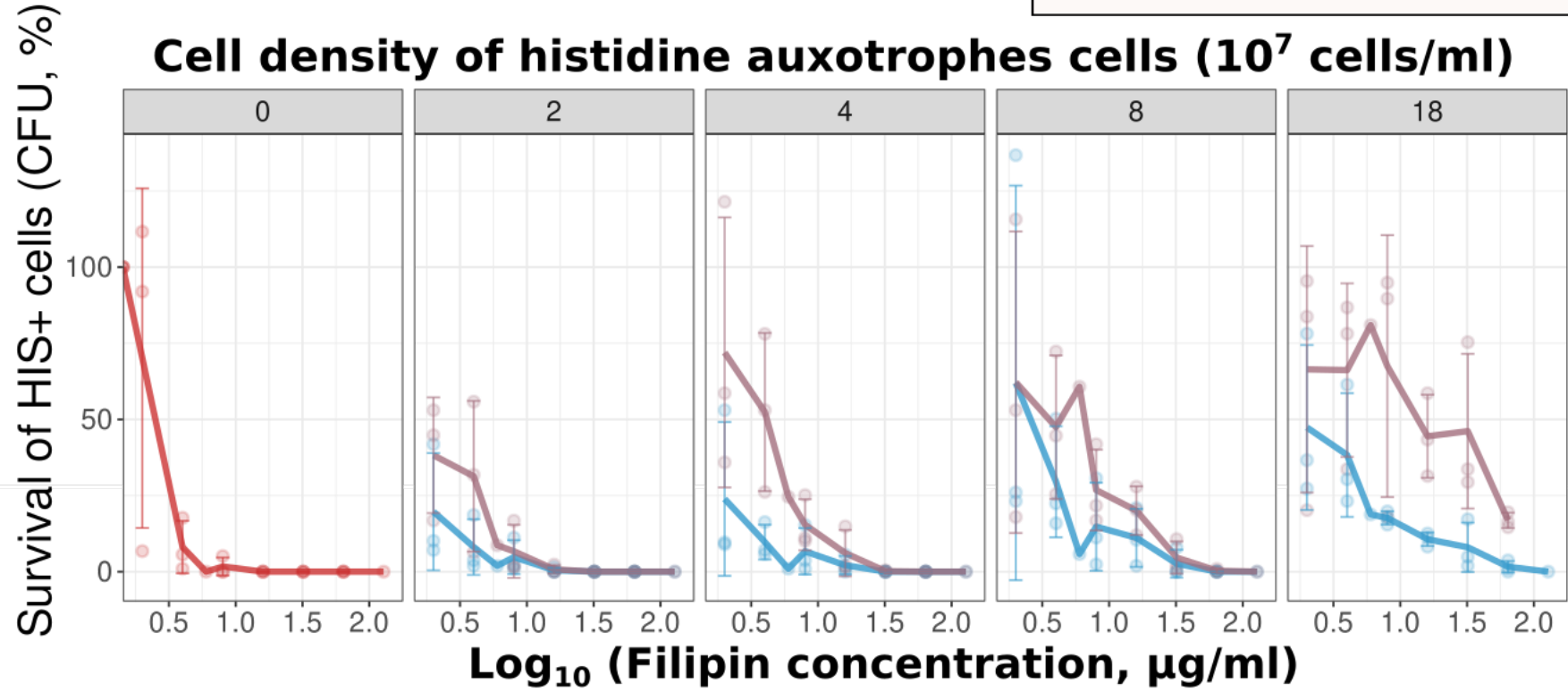

Supplement: FIG S3 [file msphere.00745-21-sf003.pdf]

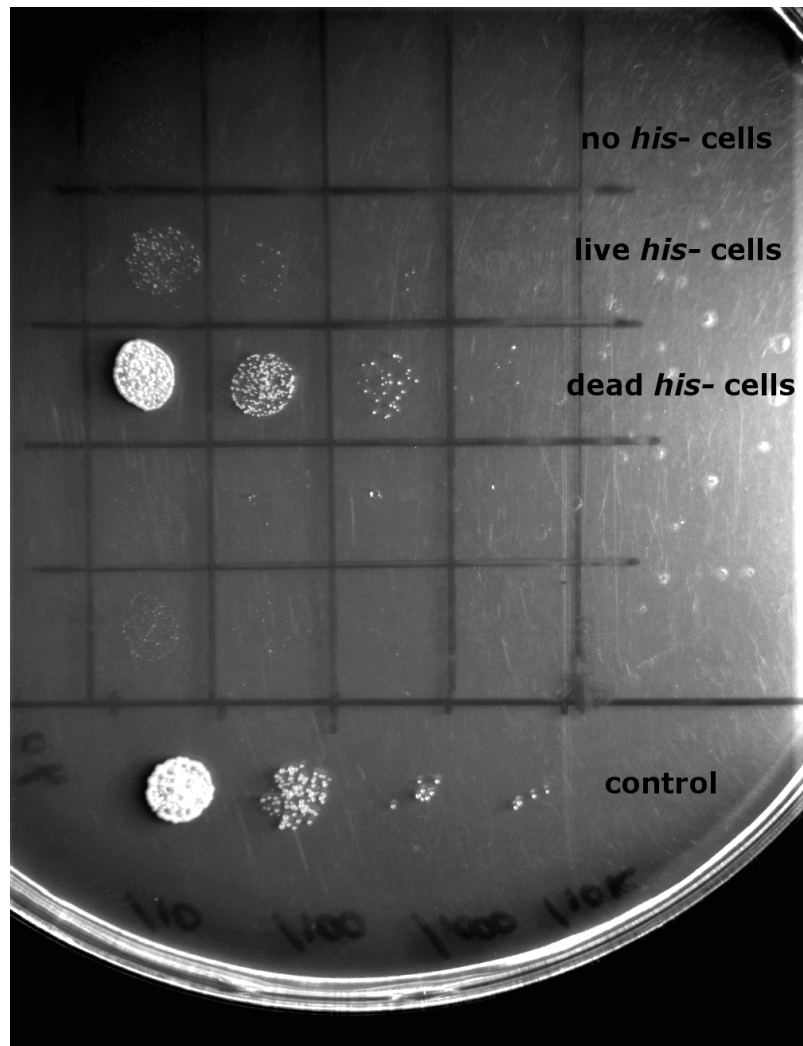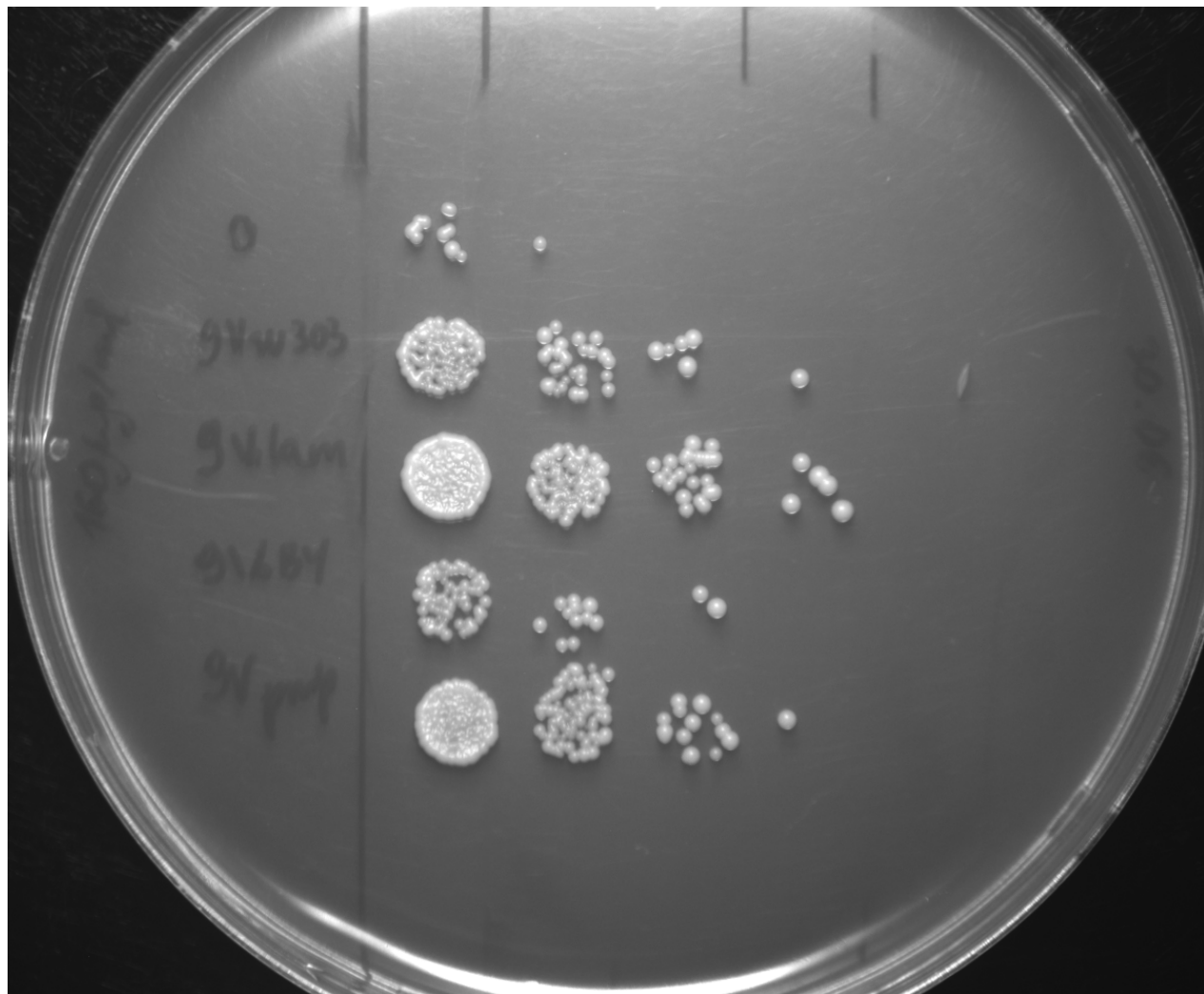

Supplement: FIG S5 [file msphere.00745-21-sf005.pdf]

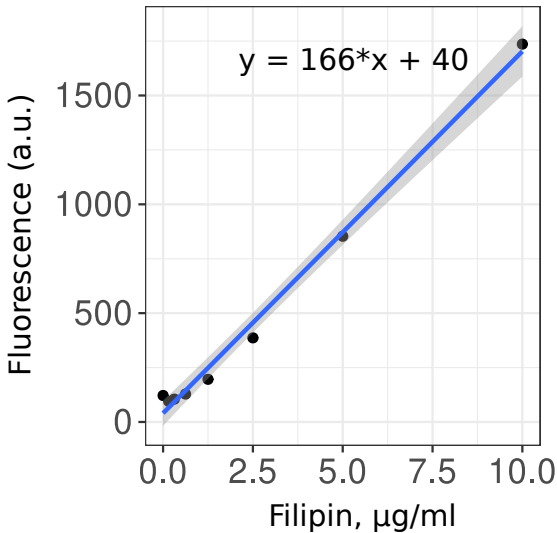

Supplement: FIG S4 [file msphere.00745-21-sf004.pdf]
