# Supplementary material for: Adaptive Role of Cell Death in Yeast Communities Stressed with Macrolide Antifungals
Source: mSphere. 2021 Nov 17;6(6):e00745-21. doi: 10.1128/mSphere.00745-21 (PMC8597739; doi:10.1128/mSphere.00745-21)
Supplement: TABLE S1 [file msphere.00745-21-st001.docx]

**Table S1. Strains used in the study**

| **Strain** | **Genotype** | **Parental strains and/or references** |
| --- | --- | --- |
| *W303-1A* | *MATa ade2-101 his3-11 trp1-1 ura3-52 can1-100 leu2-3* | Laboratory of A. Hyman |
| *HIS+* | *MATa ade2-101 his3-11 trp1-1 ura3-52 can1-100 leu2-3 HIS3* | [*(Galkina et al. 2020)*](https://paperpile.com/c/zi4Yzx/91qZ6) |
| *TRP+* | *MATa ade2-101 his3-11 trp1-1 ura3-52 can1-100 leu2-3 TRP1* | [*(Galkina et al. 2020)*](https://paperpile.com/c/zi4Yzx/91qZ6) |
| *Δlam1Δlam2Δlam3Δlam4* | *MATa ade2-101 his3-11 trp1-1 ura3-52 can1-100 leu2-3 MATa ade2-101 his3-11 trp1-1 ura3-52 can1-100 leu2-3 Δlam3::kanMX4 Δlam2::TRP1 Δlam1::NAT Δlam4::loxP* | [*(Sokolov et al. 2020)*](https://paperpile.com/c/zi4Yzx/rMsGd) |
| *BY4741* | *MATalpha his3Δ1 leu2Δ0 met15Δ0 ura3Δ0* | *Deletion collection* [*(Giaever et al. 2002)*](https://paperpile.com/c/zi4Yzx/rJVq) |
| *BY Δpmp3* | *MATalpha his3Δ1 leu2Δ0 met15Δ0 ura3Δ0 Δpmp3::kanMX4* | *Deletion collection* [*(Giaever et al. 2002)*](https://paperpile.com/c/zi4Yzx/rJVq) |
| P_GAL1_*-CTT1* | *MATa ade2-101 his3-11 trp1-1 ura3-52 can1-100 leu2-3 PGAL1-CTT1::HIS3* | *W303-1A*  *this study* |

## References

[Galkina, Kseniia V., Joseph M. Finkelberg, Olga V. Markova, Aglaia V. Azbarova, Atanu Banerjee, Sonam Kumari, Svyatoslav S. Sokolov, Fedor F. Severin, Rajendra Prasad, and Dmitry A. Knorre. 2020. “Protonophore FCCP Provides Fitness Advantage to PDR-Deficient Yeast Cells.” *Journal of Bioenergetics and Biomembranes* 52 (5): 383–95.](http://paperpile.com/b/zi4Yzx/91qZ6)

[Giaever, Guri, Angela M. Chu, Li Ni, Carla Connelly, Linda Riles, Steeve Véronneau, Sally Dow, et al. 2002. “Functional Profiling of the Saccharomyces Cerevisiae Genome.” *Nature* 418 (6896): 387–91.](http://paperpile.com/b/zi4Yzx/rJVq)

[Sokolov, Svyatoslav S., Margarita A. Vorobeva, Alexandra I. Smirnova, Ekaterina A. Smirnova, Nataliya I. Trushina, Kseniia V. Galkina, Fedor F. Severin, and Dmitry A. Knorre. 2020. “LAM Genes Contribute to Environmental Stress Tolerance but Sensibilize Yeast Cells to Azoles.” *Frontiers in Microbiology* 11 (1): 38.](http://paperpile.com/b/zi4Yzx/rMsGd)
